# Post-Control Surveillance of *Triatoma infestans* and *Triatoma sordida* with Chemically-Baited Sticky Traps

**DOI:** 10.1371/journal.pntd.0001822

**Published:** 2012-09-13

**Authors:** Antonieta Rojas de Arias, Fernando Abad-Franch, Nidia Acosta, Elsa López, Nilsa González, Eduardo Zerba, Guillermo Tarelli, Héctor Masuh

**Affiliations:** 1 Centro para el Desarrollo de la Investigación Científica, Díaz Gill Medicina Laboratorial/Fundación Moisés Bertoni, Asunción, Paraguay; 2 Instituto Leônidas e Maria Deane – Fiocruz Amazônia, Manaus, Brazil; 3 Departamento de Medicina Tropical, Instituto de Investigaciones en Ciencias de la Salud, Universidad Nacional de Asunción, Asunción, Paraguay; 4 Centro de Investigaciones de Plagas e Insecticidas, Buenos Aires, Argentina; Universidad Autónoma de Yucatán, Mexico

## Abstract

**Background:**

Chagas disease prevention critically depends on keeping houses free of triatomine vectors. Insecticide spraying is very effective, but re-infestation of treated dwellings is commonplace. Early detection-elimination of re-infestation foci is key to long-term control; however, all available vector-detection methods have low sensitivity. Chemically-baited traps are widely used in vector and pest control-surveillance systems; here, we test this approach for *Triatoma* spp. detection under field conditions in the Gran Chaco.

**Methodology/Principal Findings:**

Using a repeated-sampling approach and logistic models that explicitly take detection failures into account, we simultaneously estimate vector occurrence and detection probabilities. We then model detection probabilities (conditioned on vector occurrence) as a function of trapping system to measure the effect of chemical baits. We find a positive effect of baits after three (odds ratio [OR] 5.10; 95% confidence interval [CI_95_] 2.59–10.04) and six months (OR 2.20, CI_95_ 1.04–4.65). Detection probabilities are estimated at *p*≈0.40–0.50 for baited and at just *p*≈0.15 for control traps. Bait effect is very strong on *T. infestans* (three-month assessment: OR 12.30, CI_95_ 4.44–34.10; *p*≈0.64), whereas *T. sordida* is captured with similar frequency in baited and unbaited traps.

**Conclusions/Significance:**

Chemically-baited traps hold promise for *T. infestans* surveillance; the sensitivity of the system at detecting small re-infestation foci rises from 12.5% to 63.6% when traps are baited with semiochemicals. Accounting for imperfect detection, infestation is estimated at 26% (CI_95_ 16–40) after three and 20% (CI_95_ 11–34) after six months. In the same assessments, traps detected infestation in 14% and 8.5% of dwellings, whereas timed manual searches (the standard approach) did so in just 1.4% of dwellings only in the first survey. Since infestation rates are the main indicator used for decision-making in control programs, the approach we present may help improve *T. infestans* surveillance and control program management.

## Author Summary

*Triatoma infestans* is the main vector of Chagas disease in southern South America. Dwelling-infesting populations are controlled through insecticide-spraying campaigns; however, dwellings are often re-infested when insecticide effects wane, and this leads to the re-establishment of disease transmission. Detecting and eliminating re-infestation foci is therefore crucial to prevent new cases. Unfortunately, available vector detection methods all have low sensitivity. Here, we show that simple sticky traps baited with widely available chemicals are significantly more sensitive than either unbaited traps or active manual searches by trained staff — the standard method used in control programs. Increased trap sensitivity (about 500% higher), together with an analytical approach that takes detection failures into account, allows us to estimate dwelling infestation rates at about 20–26%; in contrast, just 0–1.4% of dwellings were identified as infested by manual searches. This large difference highlights the importance of enhancing surveillance systems, and reveals how crude infestation indices may mislead decision-makers. We conclude that chemically baited sticky traps can help improve *T. infestans* surveillance systems and thus strengthen vector control program management.

## Introduction

Chagas disease control remains a major public health challenge in Latin America, where ∼7.5 million people are estimated to be infected by *Trypanosoma cruzi*, its etiological agent [1]. *T. cruzi* is primarily transmitted by blood-sucking triatomine bugs [2]; in the absence of effective vaccines, prevention heavily relies on the control of dwelling-infesting vector populations [1], [3]. Residual insecticide spraying is generally effective, but re-infestation of treated dwellings often ensues when spraying is interrupted or triatomines develop insecticide resistance [4]. As a consequence, continuous entomological surveillance is crucial for keeping dwellings free of triatomine bugs, and thus interrupting *T. cruzi* transmission to humans, in the long run [4].

Yet, detecting re-infestation can be difficult; in post-control settings, triatomine populations tend to be small and often occupy substandard houses where the extensive availability of complex refuges makes foci even harder to detect [4], [5]. The design, development, and testing of more sensitive tools for detecting re-infestation foci is therefore one key requirement of enhanced surveillance systems [4]. This is even more important in areas where wild populations of highly competent *T. cruzi* vectors act as sources of re-infesting bugs; this is the case of *Triatoma infestans*, the most important vector of Chagas disease, in the Gran Chaco of Paraguay, Argentina, and Bolivia [6]–[9].

Chemically-baited insect traps are widely used against agricultural pests [10], [11]; they are major components of tsetse fly control in Africa [12], and have shown promise for reducing adult mosquito populations [13]. These traps use volatile semiochemicals composed of natural pheromones or synthetic molecules that mimic pheromone effects (parapheromones); semiochemicals lure insects to traps, where they are killed [10], [11], [13]. Here, we provide a field test of this approach for detecting small *T. infestans* and *T. sordida* re-infestation foci after insecticide application in the Chaco of Argentina and Paraguay. We use a modeling approach that accounts for imperfect detection and yields statistical estimates of detection probabilities, conditioned on occurrence; then, the effects of semiochemicals on such probabilities can be measured.

## Methods

### Setting

**Figure 1:**
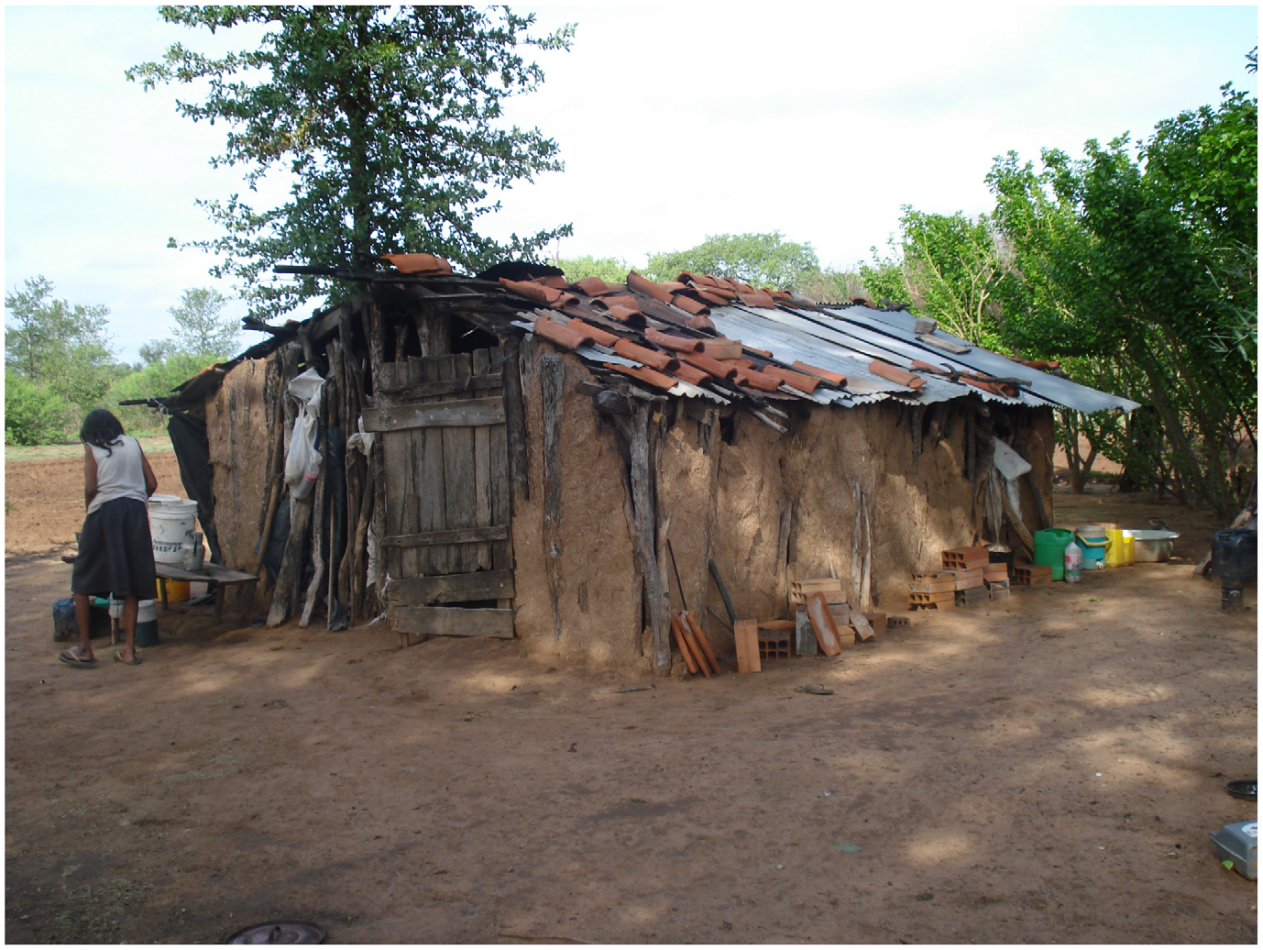
Study setting: typical indigenous dwelling from the study area in the Paraguayan Chaco.

Two rural areas with known history of high dwelling infestation rates by *T. infestans* were studied between 2004 and 2005. Both are located within the Gran Chaco biome, one in Argentina and one in Paraguay. In Paraguay, the study area (Yalve Sanga; 22°40′S, 59°45′W) lies within a semiarid region with xerophytic vegetation and long periods of drought. Average annual precipitation is 600 mm and average annual temperature 26°C. Local communities rely on subsistence farming and hunting-gathering. Most houses had mud, wood-plank or palm-stems walls, tiled or wooden roofs, and only one room; the peridomestic area typically had no firewood piles or building material heaps and very few chicken coops (Fig. 1). Our sample included 278 dwellings in Paraguay. In Argentina, the study area (Añatuya, Santiago del Estero; 28°27′S, 62°50′W) lies within the Parque Chaqueño Seco, with *quebracho* trees (*Aspidosperma* sp., *Schinopsis* sp.) occurring in forest patches interspersed within pastures and open land. Temperature varies around an annual average of ∼21.5°C, reaching 47°C in summer and dropping to −5°C in winter. Annual rainfall ranges from 500 to 950 mm. Houses typically had plastered brick walls and tiled roofs. Most dwellers reared fowl, pigs, and/or goats in the peridomestic area; coops, sties, and corrals were thus common. In total, 189 dwellings were studied in Argentina. In a pre-intervention, baseline survey, manual vector searches revealed rates of dwelling infestation by *T. infestans* of 27.3% in Paraguay and 72.9% in Argentina (where 0.5% tetramethrin was used as flushing-out agent). At this time-point, all dwellings were sprayed with λ-cyhalothrin (WP-10%) or deltamethrin (SC-2.5%) by vector control agents, and were therefore regarded as putatively non-infested at the time of trap setting one month later.

### Intervention

**Figure 2:**
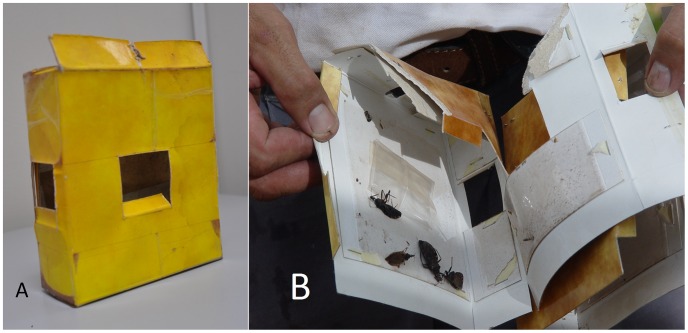
Chemically-baited sticky trap used for Chagas disease vector surveillance. (A), outer aspect; (B), triatomines caught in the entomological glue inside a trap; the bag containing the semiochemical bait is also visible.

Two traps were nailed on opposite walls (chosen haphazardly) of each house, 1.5 m above ground level; one was baited with a semiochemical and the other was an unbaited (hereafter also called ‘control’) trap. In Argentina, 21 trap pairs were placed in peridomestic structures (chicken coops, pigsties, and goat corrals), nailed to walls or trunks and protected from rain; these were considered as separate sampling units in the analyses, for a total of 210 units (which we dub ‘dwellings’ for consistency) analyzed. Traps consisted of a cardboard box (10×10×4 cm) with one opening in the back and one in each side. The boxes contained folded paper, and interior walls were covered with entomological glue (Fig. 2). Semiochemicals (Sigma-Aldrich) were placed inside traps in ∼6×6 cm, heat-sealed polyethylene sachets with 0.1 mm-thick walls; laboratory tests at the Centro de Investigaciones de Plagas e Insecticidas (CIPEIN, Argentina) showed slow Hexanal and Benzaldehyde release from polyethylene vials (see Table S1). Hexanal and Nonanal were tested in Paraguay at a dose of 200 µl/trap (185 and 93 dwellings, respectively); in Argentina, 500 µl/trap of either Octanal or Benzaldehyde were used (119 and 91 dwellings, respectively). All traps were checked for triatomines after one, three, and six months since the start of the intervention; positive traps and all baits were replaced at each assessment. Standard, 1 man*hour manual searches were conducted during each assessment; in Argentina, 0.5% tetramethrin was used as a flushing-out agent, whereas no such agent was used in Paraguay. Local vector control agents applied a synthetic pyrethroid insecticide (as above) whenever a triatomine breeding colony (i.e., with immature stages) was found in a dwelling.

### Data analysis

We use a hierarchical modeling approach based on repeated sampling of single ecotopes or habitat patches [14]–[16]; an ecotope is defined here as each dwelling in the study. The individual result of each trap is a binary variable that takes values of one when vectors are detected and zero otherwise. Thus, for each assessment and dwelling, we have a detection history consisting of either “00” (no trap detected bugs), “11” (both traps did so), “10” (only the baited trap detected vectors), or “01” (only the control trap did so). Note that, in the first case (“00”), we explicitly consider the possibility that bugs were present but went undetected [14], [15].

Since we are particularly interested in comparing the performance of different vector-detection devices, our models emphasize the sampling-process component of the hierarchy. This allows us to derive an estimate of the probability of detecting the vectors, conditioned on their occurrence (i.e., the method's sensitivity, denoted *p*); this estimate is then modeled as a function of the trapping method. The models also include a biological-process component (denoted Ψ) that expresses the probability that an ecotope is occupied by the vectors – or, equivalently, a measure of dwelling infestation rates [14]–[17]. Since these estimates of occupancy explicitly account for imperfect detection, they are expected to be less biased [14]–[17] than the standard ‘infestation index’ recommended by the World Health Organization [3].

Logistic regression models were fit via maximum likelihood (ML) using the program PRESENCE 4.0 [18]. Our approach assumes population closure (which the simultaneous assessment of both traps ensures) and independence of dwellings with regard to infestation (which may be violated in some instances but should not affect trap sensitivity estimates). Traps set in the same dwelling are also treated as independent samples with regard to vector detection in the logistic models; to check the robustness of modeling results to non-independence of traps, we complement models with McNemar odds ratios (ORs) for correlated proportions [19], [20]. Models were compared using Akaike's information criterion (AIC), which takes lower values for models with a better compromise between fit (as assessed by likelihood values) and complexity (as the number of estimable parameters) [21]. Effect sizes are reported as ML estimates of slope parameters (*β*) and/or as exp(*β*) = OR. Standard errors are always given after a “±” sign, and “CI_95_” denotes (Wald-type) 95% confidence interval limits. Note that the results of manual bug searches, which are not directly comparable with longitudinal trap results, were not included in logistic models or McNemar OR calculations.

We also carried out separate analyses for the two main vector species found during our surveys, *T. infestans* and *T. sordida*. However, low capture rates precluded the use of the modeling approach described above in species-specific appraisals for some of the assessments; in these cases, we present only McNemar ORs.

### Ethics statement

The study was approved by the Institutional Review Board of the Instituto de Investigaciones en Ciencias de la Salud, Universidad Nacional de Asunción, Paraguay (31/05/2002). In both countries, the project was discussed in community meetings; participation of local residents was optional and involved no remuneration.

## Results

One month after traps were set, the following observations were made. In Paraguay, infestation was detected by timed manual searches in four dwellings. Hexanal-baited traps were positive in one of these four dwellings and in 11 dwellings with negative manual searches; six control traps paired with Hexanal-baited ones were positive. Six Nonanal-baited traps detected infestation in dwellings where unbaited traps and manual searches were both negative. In Argentina, nine Octanal-baited and seven Benzaldehyde-baited traps identified infestation, whereas timed manual searches and unbaited traps were all negative. These initial results suggested a relatively fair performance of baited traps, but the low numbers of positive traps (particularly control traps) led us to regard them as inconclusive. We therefore restrict our inferences to the results of assessments made after three months (which we pooled with the results from the initial, one-month assessment) and six months of trap operation (Tables 1, 2, and 3).

**Table 1:** Triatomine detection using chemically-baited and unbaited sticky traps after three months of trap operation.

Country/Semiochemical				
Paraguay				
Hexanal		Baited trap ⊕	Baited trap ⊘	Total
	Unbaited trap ⊕	6•	10§	16
	Unbaited trap ⊘	15#	154¶	169
	Total	21	164	185
Nonanal		Baited trap ⊕	Baited trap ⊘	Total
	Unbaited trap ⊕	1	0	1
	Unbaited trap ⊘	11	81	92
	Total	12	81	93
Overall		Baited trap ⊕	Baited trap ⊘	Total
	Unbaited trap ⊕	7	10	17
	Unbaited trap ⊘	26	235	261
	Total	33	245	278
Argentina*				
Octanal		Baited trap ⊕	Baited trap ⊘	Total
	Unbaited trap ⊕	0	0	0
	Unbaited trap ∅	16	103	119
	Total	16	103	119
Benzaldehyde		Baited trap ⊕	Baited trap ⊘	Total
	Unbaited trap ⊕	2	0	2
	Unbaited trap ⊘	9	80	89
	Total	11	80	91
Overall		Baited trap ⊕	Baited trap ⊘	
	Unbaited trap ⊕	2	0	2
	Unbaited trap ⊘	25	183	208
	Total	27	183	210

⊕ , trap positive;

⊘ , trap negative; in each 2×2 sub-table, cells contain the number of dwellings in which both trap types.

(•) , only baited traps.

(#) , only unbaited traps.

(§) , or neither baited nor unbaited traps.

(¶) captured triatomines.

* Captures include 13 positive traps set in peridomestic structures in Argentina: six were baited with Octanal, five with Benzaldehyde, and two were unbaited. Manual searches were positive in four Paraguayan and three Argentinean dwellings at the three-month assessment; in two of them, traps were negative.

**Table 2:** Triatomine detection using chemically-baited and unbaited sticky traps after six months of trap operation.

Country/Semiochemical				
Paraguay				
Hexanal		Baited trap ⊕	Baited trap ⊘	Total
	Unbaited trap ⊕	9•	9§	18
	Unbaited trap ⊘	15#	152¶	167
	Total	24	161	185
Nonanal*		Baited trap ⊕	Baited trap ⊘	Total
	Unbaited trap ⊕	0	1	1
	Unbaited trap ⊘	7	81	88
	Total	7	82	89
Overall		Baited trap ⊕	Baited trap ⊘	Total
	Unbaited trap ⊕	9	10	19
	Unbaited trap ⊘	22	233	255
	Total	31	243	274
Argentina				
Octanal		Baited trap ⊕	Baited trap ⊘	Total
	Unbaited trap ⊕	0	0	0
	Unbaited trap ⊘	0	119	119
	Total	0	119	119
Benzaldehyde		Baited trap ⊕	Baited trap ⊘	Total
	Unbaited trap ⊕	0	0	0
	Unbaited trap ⊘	0	91	91
	Total	0	91	91
Overall		Baited trap ⊕	Baited trap ⊘	
	Unbaited trap ⊕	0	0	0
	Unbaited trap ⊘	0	210	210
	Total	0	210	210

⊕ , trap positive;

⊘ trap negative; in each 2×2 sub-table, cells contain the number of dwellings in which both trap types.

(•) , only baited traps.

(#) , only unbaited traps.

(§) , or neither baited nor unbaited traps.

(¶) captured triatomines.

* Four dwellings could not be re-assessed at this time-point. Manual searches were negative in all dwellings.

**Table 3:** Post-control surveillance of domestic infestation by *Triatoma infestans* and *T. sordida* with chemically-baited traps: bait effects estimated with hierarchical logistic models.

Assessment	Model structure	AIC	N	*k*	Effect on *p*			Effect on ψ
					*β*_bait_(SE)	β_Hexanal_(SE)	β_Nonanal_(SE)	β_Paraguay_(SE)
3-month	ψ(.), *p*(bait)	515.4	488	3	1.63(0.35)	-	-	-
	ψ(Paraguay), *p*(bait)	516.8	488	4	1.63(0.35)	-	-	0.25(0.31)
6-month	ψ(.), *p*(Hexanal,Nonanal)	356.6	484	4	-	1.78(0.42)	1.39(0.66)	-
	ψ(.), *p*(bait)	369.7	484	3	0.79(0.38)	-	-	-
6-month*	ψ(.), *p*(bait)	320.2	274	3	0.79(0.38)	-	-	-
	ψ(.), *p*(Hexanal)	320.4	274	2	-	0.81(0.39)	-	-
	ψ(.), *p*(Hexanal,Nonanal)	321.8	274	4	-	0.88(0.41)	0.46(0.63)	-

Model structure: ψ, vector occurrence probabilities; *p*, vector detection probabilities, conditioned on occurrence (in parentheses, covariate names, with “.” denoting no covariate in that part of the model). AIC, Akaike information criterion; N, number of sampling units; *k*, number of parameters; *β*, estimated slope parameter; SE, standard error.

* Paraguay data only.

### Three-month assessment

**Figure 3:**
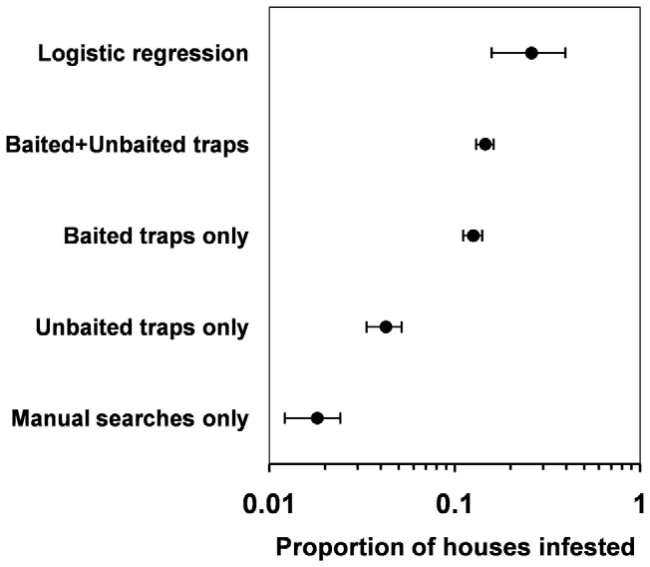
Dwelling infestation rates after three months of trap operation. Top-bottom: infestation estimate from a simple logistic regression model taking detection failures into account; infestation index after combined observations from chemically-baited and unbaited sticky traps; infestation index after observations only from chemically-baited sticky traps; infestation index after observations only from unbaited sticky traps; and infestation index after observations only from manual searches by trained staff. Solid circles are point estimates/proportions, and short vertical lines are the 95% confidence interval limits; in the case of observed proportions, these limits were estimated using the Agresti-Coull method [22]. Note that the *x*-axis is in log_10_ scale.

The results of this joint assessment (Argentina and Paraguay) are described in Table 1. Overall, they suggest a better performance of baited than control traps. A simple model comparing both trap types (first model in Table 3) shows a relatively large, positive effect of semiochemical baits on detection probabilities (*β*_bait_ = 1.63±0.35; OR 5.10, CI_95_ 2.57–10.14). This effect size estimate is nearly identical to the McNemar OR (5.10, CI_95_ 2.59–10.05), suggesting that modeling results are robust to the possible non-independence of trap pairs set within the same dwelling. The model-estimated sensitivity of baited traps (*p*_bait_ = 0.47±0.12) is over three times higher than that of control traps (*p*_control_ = 0.15±0.05). This simple model, which has no biological-process covariates (Table 3), estimates the overall infestation probability at Ψ = 0.26±0.06, nearly twice the infestation index (0.14) calculated using only observed data (from both trap types). Figure 3 provides a comparison of infestation estimates derived from this model and from crude data, including what we would be reporting had we used just one method to determine infestation; CI_95_ limits for observed proportions (as opposed to the model estimate) were calculated using the Agresti-Coull method [22].

Using an alternative model (second in Table 3), we tested for possible differences in infestation rate estimates between Paraguay and Argentina, but found no convincing evidence: the effect-size estimate was small and non-significant, and the model was less supported by the data, as assessed using AIC, than the previous, simpler one (Table 3). This alternative model, which also includes a ‘baited/unbaited’ sampling-process covariate, estimates country-specific infestation rates at Ψ_Paraguay_ = 0.28±0.07 and Ψ_Argentina_ = 0.23±0.06; infestation indices (after results from both trap types) are 0.16 and 0.13, respectively.

Finally, we ran a separate model in which each semiochemical compound entered as a sampling-process covariate. Most effect-size estimates were however very imprecise (*β*_Hexanal_ = 1.22±0.42; *β*_Nonanal_ = 3.03±2.68; *β*_Octanal_ = 3.33±3.36; *β*_Benzaldehyde_ = 2.66±1.97), indicating that more statistical power is required to detect and accurately estimate the effects of individual compounds. In addition, this model (not shown in Table 3) did not perform any better (AIC>2 units larger) than the model specifying only whether traps were baited or unbaited (first in Table 3), suggesting that the overall performance of all compounds was similar.

### Six-month assessment

Six months after traps were first set, no infestation was detected by either traps or manual searches in the Argentinean study sites. In Paraguay, triatomines were detected in 41 dwellings; in all cases, infestation was identified only with traps: timed manual searches were always negative. Baited traps again appeared to be more sensitive than control, unbaited traps (Table 2).

The model with the best compromise between fit and complexity (lowest AIC; third model in Table 3) included two sampling-process covariates describing, respectively, whether traps were baited with Hexanal or Nonanal; a model with only one (baited/unbaited) covariate (fourth in Table 3) estimates bait effect at OR = 2.20 (CI_95_ 1.04–4.65), but has an AIC value 13.13 units larger than the two-covariate model; therefore, we base inference on this latter (third model, Table 3).

The overall (Paraguay plus Argentina) observed infestation index was 0.085 (at least one trap positive in 41/484 dwellings), while the model-derived estimate was Ψ = 0.20 (CI_95_ 0.11–0.34). Both Hexanal and Nonanal had positive, significant effects on detection probabilities (Table 3). An inverse variance-weighted average [23] of these effects yields an OR = 5.30 (CI_95_ 4.14–6.77). Detection probabilities are estimated as *p*_Hexanal_ = 0.50±0.12, *p*_Nonanal_ = 0.41±0.19, and *p*_control_ = 0.15±0.05; the data did not allow for estimates regarding Benzaldehyde or Octanal, used only in Argentina.

Since no infestation was detected in Argentina, we ran a separate set of models with only data from Paraguay (*N* = 274 dwellings; last three models in Table 3). The lowest-AIC specification includes one sampling-process covariate describing whether traps were baited or unbaited. This model estimates infestation rates at Ψ = 0.24±0.05, 1.6 times higher than the observed infestation index (after results from both trap types, 0.15); vector detection probabilities, conditioned on occurrence, are estimated as *p*_bait_ = 0.47±0.11 and *p*_control_ = 0.29±0.08. Again, the semiochemical bait effect-size estimate (*β*_bait_ = 0.79±0.38; OR 2.20, CI_95_ 1.04–4.64) derived from the model (Table 3) is nearly identical to the McNemar OR (2.20, CI_95_ 1.04–4.65); albeit positive and marginally significant at the 5% level, this effect is considerably smaller than that detected in the three-month assessment.

### Species-specific results

In Paraguay, both *T. infestans* and *T. sordida* were detected by trapping; the capture of a single *T. guasayana* female was disregarded for species-specific analysis. Only *T. infestans* was captured in Argentina.

**Figure 4:**
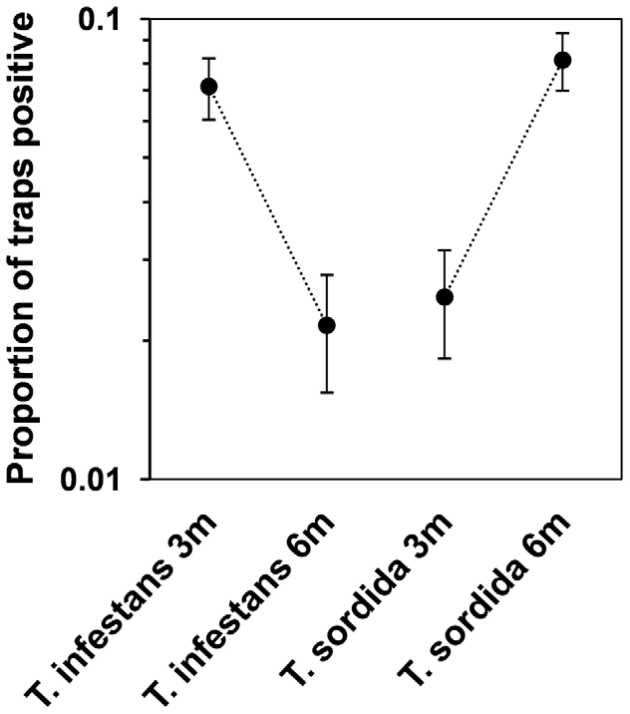
Proportion of traps that captured *Triatoma infestans* and *T. sordida* after three months (*N* = 556 traps) and six months (*N* = 548 traps) of trap operation. Solid circles are proportions; 95% confidence intervals (between short horizontal lines) were calculated using the Agresti-Coull method [22]. The sharp decline of *T. infestans* is mirrored by a similar rise of *T. sordida* captures. Note that the *y*-axis is in log_10_ scale.

Table 4 presents species-specific results for assessments conducted in Paraguay. While *T. infestans* was captured more often in baited than in unbaited traps, *T. sordida* appears to enter baited and control traps with similar frequency. Table 4 also shows that *T. infestans* was more frequently captured at the three- than at the six-month assessment, whereas *T. sordida* captures soared over the same period (Fig. 4).

**Table 4:** *Triatoma infestans* and *T. sordida* re-infestation assessment using chemically-baited and unbaited sticky traps in the Gran Chaco, Paraguay.

Species*/Assessment				McNemar
*T. infestans*				
Three-month		Baited trap ⊕	Baited trap ∅	
	Unbaited trap ⊕	6•	3§	
	Unbaited trap ∅	24#	245¶	8.00 (2.41–26.57)
Six-month		Baited trap ⊕	Baited trap ∅	
	Unbaited trap ⊕	0	2	
	Unbaited trap ∅	10	262	5.00 (1.10–22.82)
*T. sordida*				
Three-month		Baited trap ⊕	Baited trap ∅	
	Unbaited trap ⊕	1	7	
	Unbaited trap ∅	2	268	0.27 (0.06–1.38)
Six-month		Baited trap ⊕	Baited trap ∅	
	Unbaited trap ⊕	9	8	
	Unbaited trap ∅	12	245	1.50 (0.61–3.67)

* Six dead triatomines could not be identified to species level and were not included in this Table.

⊕ , trap positive;

⊘ , trap negative; in each 2×2 sub-table, cells contain the number of dwellings in which both trap types.

(•) , only baited traps.

(#) , only unbaited traps.

(§) , or neither baited nor unbaited traps.

(¶) captured triatomines.

The column named ‘McNemar’ gives, for each species and assessment, the McNemar odds ratio (95% confidence interval) for correlated proportions; to facilitate comparison with slope (*β*) estimates from logistic models, here we provide ln(odds ratio) values (95% confidence interval): *T. infestans* three-month 2.1 (0.9–3.3), six-month 1.6 (0.1–3.1); *T. sordida* three-month −1.3 (−2.8–0.3), six-month 0.4 (−0.5–1.3).

Indeed, the rarity of *T. infestans* and *T. sordida* captures in the six- and three-month assessment, respectively, restricted modeling to the pooled *T. infestans* data from the three-month assessment (Paraguay and Argentina, *N* = 488 dwellings: both traps positive, 7 dwellings; only baited traps positive, 49 dwellings; only unbaited traps positive, 4 dwellings; both traps negative, 428 dwellings). The results show a large, positive effect of baits on *T. infestans* detection (*β*_bait_ = 2.51±0.52; OR 12.30, CI_95_ 4.44–34.10); this effect is once again nearly identical to the estimated McNemar OR (12.25, CI_95_ 4.42–39.95). The observed infestation index (after results from both trap types, 0.12) is substantially lower than the estimated rate when imperfect detection is accounted for (Ψ = 0.18, CI_95_ 0.11–0.28). Trap sensitivity is estimated as *p*_bait_ = 0.64±0.15 and *p*_control_ = 0.13±0.04.

## Discussion

With the aim of helping enhance Chagas disease surveillance, we investigated the performance of a novel vector-detection system using a modeling approach developed for wildlife research and management [14], [15], [24]–[26]. This approach yields estimates of vector occurrence and detection probabilities and has several major strengths. First, one does not need to make the unrealistic assumption that a dwelling is not infested when triatomine bugs are not seen there during a survey. As our analyses demonstrate, the sensitivity of any of the methods we used is too low for a negative result to be uncritically accepted. When qualitatively comparing results from different methods, we identified a large downward bias of infestation indices derived from timed manual searches. Second, our analyses yield an ML estimate of the sensitivity of each trapping system (and with a measure of uncertainty) in the absence of results from a ‘gold-standard’ (100% sensitivity) technique. Finally, we are also able to derive a measure of bait effects including size, direction, and uncertainty. Together, these improvements should allow for informed, evidence-based decision-making in a way that standard approaches, which unrealistically assume perfect detection, do not.

Our analyses have however several limitations that must be kept in mind when interpreting the results. First, traps set in the same dwelling may not be independent; specifically, a vector entering one trap is not available for trapping in the other. In extreme cases, all bugs present in a dwelling could be caught in one trap, resulting in underestimation of the sensitivity of the paired trap and, hence, some degree of ψ overestimation [27]. In this sense, our matched-pair, same-dwelling design is a trade-off between true trap independence and reduced confounding (e.g., from spatial heterogeneity in infestation). Acknowledging this potential drawback, we used a simple yet powerful approach to estimate the effects of interest under non-independence – McNemar ORs (see ref. [20]). Model-derived estimates and McNemar ORs were virtually identical, suggesting that this potential problem was overall negligible and that our results are robust to trap dependence. Second, different semiochemicals were tested in different settings and seasons (Paraguay, July-January; Argentina, November–May), and this could confound effect-size estimates; we nonetheless found little evidence of differences between sites or among compounds. More data would be required to characterize chemical bait differences and thus identify the best-performing product. Additionally, the results of a timed manual search and a three-month trapping effort cannot be directly compared: the former may yield a negative result because no bugs were actually present in a dwelling at the time of searching. We consequently refrained from making formal quantitative comparisons. However, since timed searches are the standard method used in surveillance, it was important to provide the results from all techniques. Finally, our analyses make use of presence/absence data, thus focusing on infestation rates – the main indicator used in control program management. Modeling vector abundance (Table S2) and how it relates to detectability is a promising field for future research, but was beyond the scope of the present paper.

The pre-intervention survey revealed higher infestation rates by *T. infestans* in Argentina, suggesting that the use of a flush-out agent increased the sensitivity of manual searches [4]. Vector populations quickly declined after insecticide spraying, particularly in Argentina, but re-infestation was common in Paraguay. However, most re-infesting bugs were *T. sordida*, not *T. infestans*, and no immature specimens were trapped in Paraguay at the six-month assessment (Table S2). This indicates that, at this time point, control/surveillance activities had successfully eliminated colonization foci in both areas – but also that wild populations, the likely source of house-invading adult triatomines, are much more common in the Paraguayan than in the Argentinean study sites. Our results thus suggest that simple sticky traps can help detect (and eliminate) adventitious adult triatomines before they establish domestic breeding colonies.

Detecting residual infestation or re-infestation foci is in fact one of the major difficulties faced by Chagas disease control-surveillance programs [4], [28]. We have shown that low-cost, widely available parapheromone semiochemicals can increase the average sensitivity of adhesive traps by between ∼410% (three-month assessment, overall) and ∼120% (six-month assessment, Paraguay). Aliphatic aldehydes had been previously shown to attract *T. infestans* under experimental conditions [29]; here, we extend these observations to provide evidence that they also hold promise in real-life scenarios. The enhanced trap sensitivity we report here occurs in a context of low infestation rates and low-density vector foci – that is, in the typical post-control setting where entomological surveillance becomes key to sustainable disease prevention [2], [4], [28], [30]. As suggested by previous analyses (see ref. [4]), timed manual searches perform poorly under such circumstances; both community involvement in reporting suspect insects [4] and, as we show here, simple chemically-baited traps are significantly more effective. These three components can obviously be combined in an integrated, flexible surveillance strategy; the evidence strongly suggests that it would represent a crucial improvement over single-approach schemes.

Many alternative triatomine-detection systems have been developed and tested (reviewed in [4]). The most commonly used are simple, unbaited, non-sticky ‘sensing devices’; an inverse variance-weighted average effect-size estimate from the results in Figure 4 of ref. [4] reveals a marginally significant effect of traps when these are compared with active manual searches (*β*_traps_ = 0.33±0.14). This effect is significantly smaller than the overall bait effect we found after three months of trap operation (*β*_bait_ = 1.63±0.35), suggesting that semiochemicals could help improve trap performance in scenarios different from the one we studied.

The overall bait effect estimate was substantially smaller in the six-month assessment (*β*_bait_ = 0.79±0.38; Table 3). Species-specific analyses indicate that this apparent reduction of overall sensitivity was most likely due to the decline of *T. infestans* populations in the six-month assessment (Fig. 4, Table S2); as shown in Table 4, this species, but not *T. sordida*, is differentially attracted by baited traps. When *T. infestans* data are analyzed separately, the bait effect is indeed much stronger, reaching a mean OR estimate of 12.30 (CI_95_ 4.44–34.10) in the three-month assessment; trap sensitivity is ∼5 times (∼500%) higher when traps are baited with semiochemicals. At six months, semiochemicals retain a significant effect, even if somewhat weaker (and with a larger CI_95_), on *T. infestans*. On the contrary, *T. sordida* are captured with roughly the same probability in baited and unbaited traps (Table 4); re-infestation by this species was frequent in our Paraguayan study sites (Fig. 4), where most trapped specimens were adults (Table S2).

As with our semiochemicals, yeast cultures (*Saccharomyces* sp.), which release CO_2_ and thus attract triatomines [31], [32], appear to have species-specific effects [33], [34]. Live-baited sticky traps (Noireau traps [35] and variations thereof) can attract a wide variety of species, and have proven useful for the study of wild triatomine populations [6], [35]–[38]; however, the logistics of handling live animals (usually mice or chicks) in the field can be demanding – a problem that is avoided with the use of chemical baits. Further research is needed to determine whether semiochemicals can enhance trap performance in the diverse ecotopes occupied by wild triatomines [2]. A recent study, for instance, shows that a multimodal artificial bait combining CO_2_, heat, and semiochemicals yields results similar to those of mice-baited traps [39]. In Chile, CO_2_-baited traps (either as dry ice or a yeast culture) have been used for sampling *T. infestans* and *Mepraia spinolai* in wild environments [40].

Could chemically-baited sticky traps be used for direct vector control? A recent paper examines through modeling the potential role of lethal traps in the control of *T. dimidiata* [41]. The results suggest that the capacity of traps to lure vectors is an important parameter determining the efficacy of the approach; however, the number of highly attractive traps needed to reach acceptable levels of vector control seems far too large for this method to be practical or cost-effective [41]. This indicates that baited traps should be best used as part of an integrated control-surveillance strategy; their usefulness would mainly depend on their sensitivity at detecting infestation, and not on their capacity to reduce vector populations by themselves. Our results represent an encouraging, major step in this direction.

### Conclusions

We have presented the first large field trial of chemically-baited sticky traps in a real-life Chagas disease vector control-surveillance setting. Using an analytical approach that explicitly accounts for imperfect detection, we have shown that, regardless of the method used, crude dwelling infestation indices systematically underestimate actual rates. By providing more reliable estimates, our approach can critically improve decision-making in the context of vector control program management. Furthermore, we have shown how widely accessible semiochemicals boost *T. infestans* detection probabilities when used to bait simple sticky traps. Combined, these findings underscore the need for enhanced entomological surveillance strategies, which would very likely benefit from integrating active searches, chemically-baited traps, and community involvement [4], [28], [30], [42].

Finally, our approach has direct applications in other areas of epidemiological research [16], [17], [43]: repeated-sampling results from vector or pathogen surveys can be used to model the effects of biological-process factors, which modulate occurrence probabilities, and sampling-process factors, which affect our ability to detect the target organism. Both types of factors must be taken into account when strong inference is aimed for [15], [24].

## Supporting Information

Table S1 **Semiochemical release system.** Release rates of hexanal and benzaldehyde from polyethylene vials (0.9 mm-thick walls) as the % of product remaining after 0 to 20 days and with varying initial loads (50, 100, and 200 µl) and temperatures (20 or 27°C) [S1]. In the field trials, heat-sealed polyethylene sachets with 0.1 mm-thick walls and loaded with either 200 or 500 µl of semiochemicals were used instead of the more costly vials. Reference S1. Cork A, Zerba E, Camps Diez F, Rojas de Arias A. Development of an odour-baited trapping system for control of the vector of Chagas disease, *Triatoma infestans*. First and Second Annual Reports. Inco DC: International Cooperation with Developing Countries (1994–1998). Contract number ERB18*CT980356
(DOC)

Table S2 **Number of bugs caught in chemically-baited and unbaited sticky traps and by timed manual searches in two areas of the Gran Chaco (Argentina and Paraguay); results are broken down by species, sex/stage, and the two assessments conducted after three and six months of trap operation.** Note the decline of *Triatoma infestans* populations and the parallel increase of *T. sordida* catches, which is mainly represented by adult bugs
(DOC)
