# Correction: Post-Control Surveillance of *Triatoma infestans* and *Triatoma sordida* with Chemically-Baited Sticky Traps

**DOI:** 10.1371/annotation/f9e422fc-25c1-45dc-a558-35763a944c0d

**Published:** 2012-12-19

**Authors:** Antonieta Rojas de Arias, Fernando Abad-Franch, Nidia Acosta, Elsa López, Nilsa González, Eduardo Zerba, Guillermo Tarelli, Héctor Masuh

In Table 2, the table's symbol legends at the bottom are misaligned. Please see the corrected Table 2 here:

http://www.plosntd.org/corrections/pntd.0001822.t002.cn.tif

