# Correction: Post-Control Surveillance of *Triatoma infestans* and *Triatoma sordida* with Chemically-Baited Sticky Traps

**DOI:** 10.1371/annotation/b5fe70a2-b190-496c-80b4-53e2aa4885d2

**Published:** 2012-12-19

**Authors:** Antonieta Rojas de Arias, Fernando Abad-Franch, Nidia Acosta, Elsa López, Nilsa González, Eduardo Zerba, Guillermo Tarelli, Héctor Masuh

In Table 1, the symbols that denote the traps were negative have accidentally been omitted. Furthermore, the table's symbol legends at the bottom are misaligned. Please see the corrected Table 1 here:

http://www.plosntd.org/corrections/pntd.0001822.t001.cn.tif

