# Supplementary material for: Post-Control Surveillance of Triatoma infestans and Triatoma sordida with Chemically-Baited Sticky Traps
Source: PLoS Negl Trop Dis. 2012 Sep 13;6(9):e1822. doi: 10.1371/journal.pntd.0001822 (PMC3441417; doi:10.1371/journal.pntd.0001822)
Supplement: Table S1 — Semiochemical release system. Release rates of hexanal and benzaldehyde from polyethylene vials (0.9 mm-thick walls) as the % of product remaining after 0 to 20 days and with varying initial loads (50, 100, and 200 µl) and temperatures (20 or 27°C) [S1]. In the field trials, heat-sealed polyethylene sachets with 0.1 mm-thick walls and loaded with either 200 or 500 µl of semiochemicals were used instead of the more costly vials. Reference S1. Cork A, Zerba E, Camps Diez F, Rojas de Arias A. Development of an odour-baited trapping system for control of the vector of Chagas disease, Triatoma infestans. First and Second Annual Reports. Inco DC: International Cooperation with Developing Countries (1994–1998). Contract number ERB18*CT980356 (DOC) [file pntd.0001822.s001.doc]

**Supporting Information**

**Table S1.** Semiochemical release system. Release rates of hexanal and benzaldehyde from polyethylene vials (0.9mm-thick walls) as the % of product remaining after 0 to 20 days and with varying initial loads (50, 100, and 200μl) and temperatures (20 or 27°C) [S1]. In the field trials, heat-sealed polyethylene sachets with 0.1mm-thick walls and loaded with either 200 or 500μl of semiochemicals were used instead of the more costly vials

| Days | Hexanal | | | | | | Benzaldehyde | | | | | |
| --- | --- | --- | --- | --- | --- | --- | --- | --- | --- | --- | --- | --- |
|  | 50μl | | 100μl | | 200μl | | 50μl | | 100μl | | 200μl | |
|  | 20°C | 27°C | 20°C | 27°C | 20°C | 27°C | 20°C | 27°C | 20°C | 27°C | 20°C | 27°C |
| 0 | 100 | 100 | 100 | 100 | 100 | 100 | 100 | 100 | 100 | 100 | 100 | 100 |
| 2 | 98.0 | 95.9 | 99.1 | 97.7 | 100 | 98.9 | 100 | 98.5 | 99.8 | 99.1 | 100 | 99.0 |
| 6 | 88.2 | 79.8 | 93.3 | 87.7 | 96.6 | 93.8 | 100 | 87.1 | 98.0 | 94.1 | 100 | 94.9 |
| 9 | 82.7 | 69.8 | 88.0 | 80.1 | 93.4 | 89.4 | 97.4 | 79.3 | 96.3 | 89.8 | 99.2 | 92.1 |
| 15 | 66.6 | 54.8 | 78.0 | 66.4 | 87.2 | 81.1 | 89.8 | 58.1 | 89.9 | 80.5 | 95.7 | 86.3 |
| 20 | 59.2 | 49.8 | 71.2 | 59.3 | 81.9 | 75.1 | 83.7 | 53.6 | 85.1 | 73.5 | 91.5 | 81.9 |

**Reference**

S1. Cork A, Zerba E, Camps Diez F, Rojas de Arias A. Development of an odour-baited trapping system for control of the vector of Chagas disease, *Triatoma infestans*. First and Second Annual Reports. Inco DC: International Cooperation with Developing Countries (1994-1998). Contract number ERB18*CT980356.
