# Supplementary material for: Post-Control Surveillance of Triatoma infestans and Triatoma sordida with Chemically-Baited Sticky Traps
Source: PLoS Negl Trop Dis. 2012 Sep 13;6(9):e1822. doi: 10.1371/journal.pntd.0001822 (PMC3441417; doi:10.1371/journal.pntd.0001822)
Supplement: Table S2 — Number of bugs caught in chemically-baited and unbaited sticky traps and by timed manual searches in two areas of the Gran Chaco (Argentina and Paraguay); results are broken down by species, sex/stage, and the two assessments conducted after three and six months of trap operation. Note the decline of Triatoma infestans populations and the parallel increase of T. sordida catches, which is mainly represented by adult bugs (DOC) [file pntd.0001822.s002.doc]

**Supporting Information**

**Table S2.** Number of bugs caught in chemically-baited and unbaited sticky traps and by timed manual searches in two areas of the Gran Chaco (Argentina and Paraguay); results are broken down by species, sex/stage, and the two assessments conducted after three and six months of trap operation. Note the decline of *Triatoma infestans* populations and the parallel increase of *T. sordida* catches, which is mainly represented by adult bugs

| Assessment | Method | Paraguay | | | | | | Argentina | |
| --- | --- | --- | --- | --- | --- | --- | --- | --- | --- |
|  |  | *T. infestans* | | | *T. sordida* | | | *T. infestans* | |
|  |  | Male | Female | Nymph | Male | Female | Nymph | Adult* | Nymph |
| Three-month | Baited | 14 | 23 | 10 | 3 | 1 | 1 | 49 | 59 |
|  | Unbaited | 5 | 9 | 15 | 2 | 8 | 0 | 5 | 3 |
|  | Manual | 0 | 5 | 5 | 0 | 0 | 0 | 2 | 3 |
|  | Sub-total | 19 | 37 | 30 | 5 | 9 | 1 | 56 | 65 |
|  | Total | 86 | | | 15 | | | 121 | |
| Six-month** | Baited | 2 | 3 | 0 | 1 | 19 | 0 | 0 | 0 |
|  | Unbaited | 1 | 2 | 0 | 3 | 16 | 0 | 0 | 0 |
|  | Manual | 0 | 0 | 0 | 0 | 0 | 0 | 0 | 0 |
|  | Sub-total | 3 | 5 | 0 | 4 | 35 | 0 | 0 | 0 |
|  | Total | 8 | | | 39 | | | 0 | |
| Grand total |  | 94 | | | 54 | | | 121 | |

*T.*, *Triatoma*; *the sex of adult bugs was not recorded in Argentina; **Six dead triatomines could not be identified to species level and were not included in this Table.
